# Serum Mac-2 binding protein glycosylation isomer and galectin-3 levels in adult-onset Still’s disease and their association with cytokines

**DOI:** 10.3389/fimmu.2024.1385654

**Published:** 2024-04-22

**Authors:** Shuhei Yoshida, Tomohiro Koga, Yuya Fujita, Hiroshi Yatsuhashi, Haruki Matsumoto, Yuya Sumichika, Kenji Saito, Shuzo Sato, Tomoyuki Asano, Masao Kobayakawa, Hiromasa Ohira, Masashi Mizokami, Masaya Sugiyama, Kiyoshi Migita

**Affiliations:** ^1^ Department of Rheumatology, Fukushima Medical University School of Medicine, 1 Hikarigaoka, Fukushima, Japan; ^2^ Department of Immunology and Rheumatology, Division of Advanced Preventive Medical Sciences, Nagasaki University Graduate School of Biomedical Sciences, Nagasaki, Japan; ^3^ Department of Hepatology, National Hospital Organization Nagasaki Medical Center, Nagasaki, Japan; ^4^ Department of Endoscopy, Fukushima Medical University Hospital, Fukushima, Japan; ^5^ Medical Research Center, Fukushima Medical University, Fukushima, Japan; ^6^ Department of Gastroenterology, Fukushima Medical University School of Medicine, Fukushima, Japan; ^7^ Genome Medical Sciences Project, National Center for Global Health and Medicine, Chiba, Japan; ^8^ Department of Viral Pathogenesis and Controls, National Center for Global Health and Medicine, Chiba, Japan

**Keywords:** adult-onset Still’s disease, cytokine, galectin-3, Mac-2 binding protein glycosylation isomer, interleukin-18, macrophage activation syndrome

## Abstract

**Background:**

Autoinflammation with cytokine dysregulation may be implicated in the pathophysiology of adult-onset Still’s disease (AOSD); however, the relationship between galectins and cytokines in patients with active AOSD remains unknown. We aimed to examine the relationship between circulating cytokines/chemokines and galectin-3 (Gal-3) or its ligand, Mac-2 binding protein glycosylation isomer (M2BPGi), in Japanese patients with AOSD.

**Methods:**

We recruited 44 consecutive patients diagnosed with AOSD according to the Yamaguchi criteria, 50 patients with rheumatoid arthritis (RA) as disease controls, and 27 healthy participants. Serum M2BPGi levels were directly measured using a HISCL M2BPGi reagent kit and an automatic immunoanalyzer (HISCL-5000). Serum Gal-3 concentrations were measured by enzyme-linked immunosorbent assay. The serum levels of 69 cytokines were analyzed in patients with AOSD using a multi-suspension cytokine array. We performed a cluster analysis of each cytokine expressed in patients with AOSD to identify specific molecular networks.

**Results:**

Significant increases in the serum concentrations of Gal-3 and M2BPGi were found in the serum of patients with AOSD compared with patients with RA and healthy participants (both p <0.001). There were significant positive correlations between serum Gal-3 levels and AOSD disease activity score (Pouchot score, r=0.66, p <0.001) and serum ferritin levels. However, no significant correlations were observed between serum M2BPGi levels and AOSD disease activity scores (Pouchot score, r = 0.32, p = 0.06) or serum ferritin levels. Furthermore, significant correlations were observed between the serum levels of Gal-3 and various inflammatory cytokines, including interleukin-18, in patients with AOSD. Immunosuppressive treatment in patients with AOSD significantly reduced serum Gal-3 and M2BPGi levels (p = 0.03 and 0.004, respectively).

**Conclusions:**

Although both Gal-3 and M2BPGi were elevated in patients with AOSD, only Gal-3 was a useful biomarker for predicting disease activity in AOSD. Our findings suggest that circulating Gal-3 reflects the inflammatory component of AOSD, which corresponds to proinflammatory cytokine induction through inflammasome activation cascades.

## 1 Introduction

Mac-2 binding protein (M2BP, also known as galectin-3 [Gal-3]-binding protein) is a highly glycosylated glycoprotein secreted and acts as a ligand of Gal-3 (formerly known as Mac-2) (1). Recently, *Wisteria floribunda* agglutinin (WFA)^+^-M2BP, which detects changes in the glycans on the surface of M2BP, was introduced as a reliable glycobiomarker for liver fibrosis. The M2BP glycosylation isomer (M2BPGi) functions as a messenger between these cells and Kupffer cells *via* Mac-2 (Gal-3), and is secreted by hepatic stellate cells during hepatic fibrosis progression (2). M2BPGi has been hypothesized to contribute to the inflammatory process and is elevated in several chronic inflammatory diseases (3). For example, serum WFA^+^-M2BP was shown to be a potential biomarker predicting the progression of fibrosis in patients with idiopathic pulmonary fibrosis (4). We also previously reported that M2BPGi is elevated in patients with autoimmune hepatitis and demonstrated a close relationship between serum levels of M2BPGi and immune-mediated hepatic inflammation, in addition to liver fibrosis, in patients with autoimmune hepatitis (5, 6). Although the functions of M2BP are not yet well defined, it interacts with Gal-3 and extracellular proteins, such as fibronectin suggesting that M2BP possess immune-activating functions (7). Additionally, Gal-3 is postulated to activate immune cells, including monocytes and macrophages (8). It has also been demonstrated that epithelial cell-associated Gal-3 activates a variety of innate immune cells to produce proinflammatory cytokines (9).

Adult-onset Still’s disease (AOSD) is a systemic autoinflammatory disease characterized by skin rash, spiking fever, arthritis, sore throat, lymphadenopathy, and hepatosplenomegaly (10). Inflammatory cytokines contribute to the development of a cytokine storm in severe AOSD (11). Interleukin (IL)-1, IL-6, and IL-18 appear to contribute to these inflammatory processes in patients with AOSD (12). Given the activating properties of M2BPGi and Gal-3 against the innate immune system, we hypothesized that these molecules might be involved in the inflammatory process of AOSD. To test this hypothesis, we aimed to determine whether serum levels of Gal-3 or M2BPGi are linked to the inflammatory processes of AOSD. We also evaluated the clinical relevance of these molecules and proinflammatory cytokines in patients with AOSD.

## 2 Materials and methods

### 2.1 Patients and study design

Our dataset comprised 44 untreated patients with AOSD, 50 patients with rheumatoid arthritis (RA) as disease controls, and 27 healthy participants. All patients diagnosed with AOSD at the Department of Rheumatology, Fukushima Medical University Hospital from January 2005 to August 2022 were enrolled. All records were accessed between January 2020 and October 2022. Patients had to be aged ≥18 years to be diagnosed with AOSD according to the diagnostic criteria developed by Yamaguchi et al. (13) after exclusion of those with infectious, neoplastic, and autoimmune disorders. Inclusion criteria were: age ≥18 years and a diagnosis of AOSD according to Yamaguchi’s diagnostic criteria (13). We collected pretreatment serum samples from the 44 patients with AOSD included in the study. Additionally, to examine longitudinal variations, we also collected serum samples from 16 patients before and after treatment. AOSD activity status was assessed by the Pouchot score (14). According to the disease course, patients were divided into three groups as described by Cush et al. (15) based on the three classic clinical types (polycyclic systemic, monocyclic, and chronic articular). Macrophage activation syndrome (MAS) was diagnosed according to the European League Against Rheumatism (EULAR)/American College of Rheumatology (ACR) guidelines including identification of hemophagocytic lymphohistiocytosis (16) and the reactive hemophagocytic syndrome diagnostic score (H score) proposed by Fardet et al. (17). Patients with RA were recruited from among those who visited the Department of Rheumatology, Fukushima Medical University, between June 2009 and April 2023. All participants met the 2010 ACR/EULAR classification criteria for the disease (18). Serum samples were collected from patients having RA with RA disease activity (Disease Activity Score [DAS] 28; C-reactive protein [CRP] >2.3) with or without treatment induction. Healthy participants were recruited from the staff of the Department of Rheumatology at Fukushima Medical University between April 2021 and April 2023, and included 14 men and 13 women between the ages of 21 and 60 years, with no illnesses under treatment.

Patients with AOSD who attended our institution between January 2005 and August 2022 and who were attending as of 2023 provided written informed consent to participate. This study was approved by the Institutional Review Board of Fukushima Medical University (No. 2021-290) and the National Center for Global Health and Medicine (NCGM-187).

### 2.2 Chemokine and cytokine measurements

The Bio-Plex 3D system (Bio-Rad, Hercules, CA, USA) was used to analyze the multiplex assay of humoral factors in accordance with the manufacturer’s guidelines. Briefly, serum samples were assessed using a Bio-Rad 3D system and a Bio-Plex Pro Wash Station equipped with a magnetic manifold (19). We obtained cytokine levels in the samples based on the standard curve for each assay plate and expressed them as serum cytokines/chemokines (pg/mL). The analysis included the use of the Bio-Plex Pro human cytokine screening 48-Plex and Bio-Plex Pro human chemokine screening 40-Plex. Additionally, C-C motif ligand (CCL)17 and interferon (IFN)-λ3 were measured using the HISCL-5000 analyzer (19) (Sysmex Corporation, Kobe, Japan). Furthermore, 69 humoral factors were measured through this assay, They included IL-1α, IL-1β, IL-1Ra, IL-2, IL-2Ra, IL-3, IL-4, IL-5, IL-6, IL-7, IL-8/CXCL8, IL-9, IL-10, IL-12(p40), IL-12(p70), IL-13, IL-15, IL-16, IL-17, IL-18, 6Ckine/chemokine CCL21, B cell-attracting chemokine-1/chemokine (C-X-C motif) ligand (CXCL) 13, cutaneous T cell-attracting chemokine CCL27, epithelial-derived neutrophil-activating protein-78/CXCL5, eotaxin/CCL11, eotaxin-2/CCL24, eotaxin-3/CCL26, fractalkine/CX3CR1-ligand, granulocyte chemotactic protein-2/CXCL6, granulocyte colony-stimulating factor (CSF), granulocyte macrophage CSF, macrophage CSF (M-CSF), growth-regulated protein-α/CXCL1, growth-regulated protein-β/CXCL2, IFN-α2, IFN-γ, IFN-λ3, I-309/CCL1, IFN-inducible T cell alpha chemoattractant/CXCL11, interferon γ-induced protein-10/CXCL10, monocyte chemotactic protein (MCP)-1/CCL2, MCP-2/CCL8, MCP-3/CCL7, MCP-4/CCL13, macrophage-derived chemokine/CCL22, macrophage migration inhibitory factor, monokine induced by interferon-γ/CXCL9, macrophage inflammatory protein (MIP)-1α/CCL3, MIP-1δ/CCL15, MIP-3α/CCL20, MIP-3β/CCL19, myeloid progenitor inhibitor factor-1/CCL23, small-inducible cytokine B16/CXCL16, stromal derived factor-1α+β/CXCL12, thymus-expressed chemokine/CCL25, tumor necrosis factor (TNF)-α, TNF-β, basic fibroblast growth factor (FGF), hepatocyte growth factor, leukemia inhibitory factor (LIF), MIP-1β, platelet derived growth factor-BB, regulated on activation, normal T cell expressed and secreted, stem cell factor, stem cell growth factor-β, TNF-related apoptosis-inducing ligand, vascular endothelial growth factor, β-nerve growth factor, and thymus and activation-regulated chemokine/CCL17.

### 2.3 Measurement of M2BPGi

Serum M2BPGi level was directly measured with the HISCL™ M2BPGi™ reagent kit (Sysmex Corporation) using an automatic immunoanalyzer HISCL‐5000 (Sysmex, Hyogo, Japan). M2BPGi levels were indexed using the following equation: cutoff index = ([M2BPGi]sample–[M2BPGi]NC)/([M2BPGi]PC)–[M2BPGi]NC), where [M2BPGi]sample represents the M2BPGi count of the serum sample, PC is the positive control, and NC is the negative control (20).

### 2.4 ELISA methods

Serum levels of Gal-3 were assessed using a Human Galectin-3 Quantikine ELISA kit (R&D Systems, Minneapolis, MN, USA) following the manufacturer’s instructions. The detection limit of Gal-3 with this ELISA kit was greater than 0.085 ng/mL.

### 2.5 Statistical analysis

Continuous variable data are expressed as median and interquartile range (IQR), while qualitative variable data are presented as frequency and percentage. Group differences among AOSD, RA, and healthy participants were analyzed using the Mann–Whitney U test. Wilcoxon signed-rank tests were used to assess the pre-and post-treatment differences. Correlations between pairs of serum markers were calculated using Spearman’s rank correlation test. Statistical analyses were conducted using the R software version 4.3.1 (R Foundation for Statistical Computing, Vienna, Austria) and SPSS version 29.0 (IBM Corp., Armonk, NY, USA). All reported p-values were two-sided, and p<0.05 was considered statistically significant. The Bonferroni correction was applied to tests for comparisons of the three groups of AOSD, RA, and healthy participants or the three disease types of AOSD, and p <0.0167 was considered significant. Additionally, Bonferroni’s correction was also applied to the multiple cytokine test (n = 69), with p <0.001 deemed significant, maintaining an overall alpha error ≤0.1.

## 3 Results

### 3.1 Demographic data of patients with AOSD

This study enrolled 44 patients with AOSD and 50 patients with RA who had a confirmed diagnosis based on the diagnostic criteria. The median age of the participants was 47 years (interquartile range [IQR] 30–62 years), and 30 were female. There were no patients with AOSD and comorbid chronic kidney disease, chronic heart disease, or liver cirrhosis. **Table 1** provides a detailed overview of the characteristics and clinical presentations of the patients with AOSD. The distribution of clinical types was as follows: polycyclic systemic in 26 patients (59.1%), monocyclic in 14 (31.8%), and chronic articular in 4 (9.1%). Additionally, the participants had a median Pouchot disease activity score of 3 (IQR 2–5). The baseline patient demographics and clinical characteristics of the 50 patients with RA were as follows. The 50 patients with RA included 29 (58.0%) female individuals (median age: 66 years, IQR: 61.3–73.5 years). Chronic kidney disease and chronic heart disease were each seen in five (10%) patients with RA. There were no patients with RA and comorbid liver cirrhosis. Most patients with RA were taking disease-modifying anti-rheumatic drugs, mostly methotrexate (30/50, 60.0%) and biologics (18/50, 36.0%). The median DAS28-CRP was 3.07 (IQR; 2.45–3.98).

**Table 1:** Demographic and clinical characteristics of patients with untreated AOSD at the time of initial diagnosis.

Variables	n=44
Female, n (%)	30 (68.2)
Age at AOSD diagnosis (years), median (IQR)	47 (30–62)
Ferritin (ng/mL), median (IQR)	2,556 (471–7,588)
CRP (mg/L), median (IQR)	51 (28.8–102.8)
WBC (/μL), median (IQR)	10,550 (8,050–14,025)
AST (U/L), median (IQR)	87 (54–187)
ALT (U/L), median (IQR)	33 (25–83)
Chronic kidney disease, n (%)	0 (0.0)
Chronic heart failure, n (%)	0 (0.0)
Liver cirrhosis, n (%)	0 (0.0)
Monocyclic type, n (%)	14 (31.8)
Polycyclic systemic type, n (%)	26 (59.1)
Chronic articular type, n (%)	4 (9.1)
Pouchot score, median (IQR)	3 (2–5)

All data are expressed as median (IQR), or numbers (percentages).

IQR, interquartile range; AOSD, adult-onset Still’s disease; CRP, C-reactive protein; WBC, white blood cell; AST, aspartate aminotransferase; ALT, alanine aminotransferase.

### 3.2 Comparison of Gal-3 and M2BPGi in patients with AOSD or RA and healthy participants

**Figure 1:**
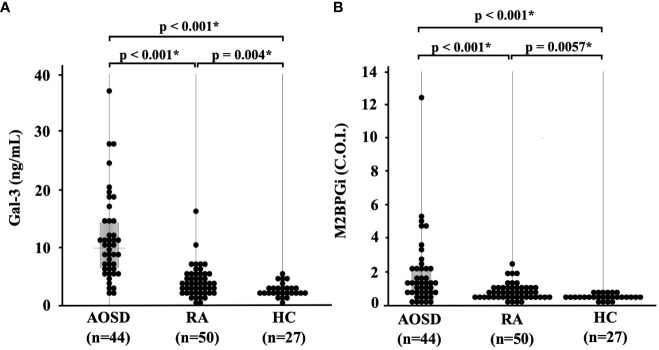
Comparison of serum **(A)** Gal-3 and **(B)** M2BPGi concentrations in AOSD patients, RA patients, and healthy participants.. Comparisons of serum Gal-3 and Gal-3BP levels in AOSD patients (n=44), RA patients (n=50) and HCs (n=27) are shown. Serum Gal-3 and M2BPGi levels are significantly higher in AOSD patients compared to those in RA patients or HCs. Gal-3, galectin-3; M2BPGi, Mac-2 binding protein glycosylation isomer; C.O.I., cut off index; AOSD, adult-onset Still’s disease; RA, rheumatoid arthritis; HC, healthy control. * means there is a significant difference at p <0.05.

**Figure 2:**
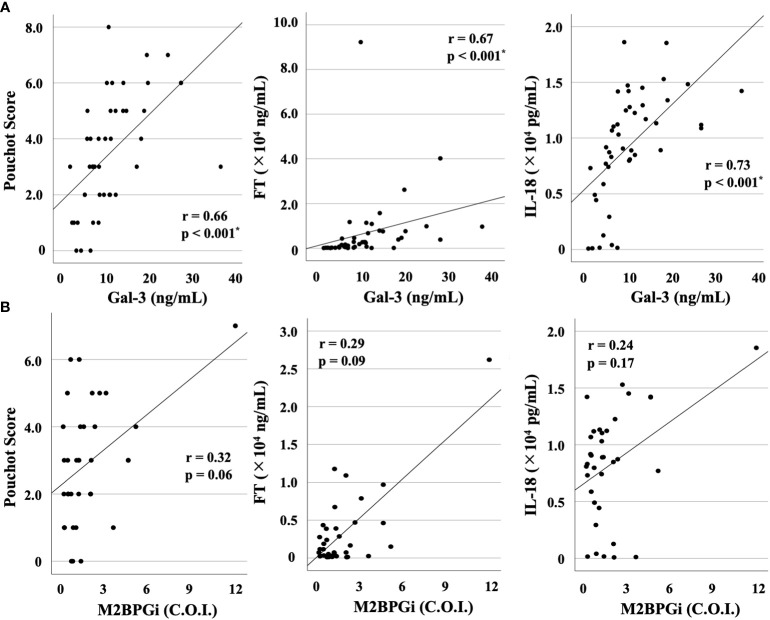
Correlations between serum levels of Gal-3 or M2BPGi and disease activity score (Pouchot score) or serum levels of FT or IL-18 in patients with AOSD. Correlations between serum levels of **(A)** Gal-3 or **(B)** M2BPGi and AOSD disease activity score, serum levels of FT and IL-18 in AOSD patients are shown. Serum levels of Gal-3 show a significant and positive correlation with Pouchot score and serum levels of FT and IL-18. Gal-3, galectin-3; M2BPGi, Mac-2 binding protein glycosylation isomer; FT, ferritin; IL, interleukin; AOSD, adult-onset Still’s disease; C.O.I., cut off index. * means there is a significant difference at p <0.05.

We compared the baseline serum levels of Gal-3 (**Figure 1A**) and M2BPGi (**Figure 1B**) among patients with AOSD or RA and healthy participants. Significant increases in the serum concentrations of Gal-3 and M2BPGi were found in the serum of patients with AOSD compared with those with RA (p <0.001 for both Gal-3 and M2BPGi) as well as with healthy participants (p <0.001 for both Gal-3 and M2BPGi). We also examined the correlation of serum Gal-3 or M2BPGi levels with the AOSD disease activity score, IL-18, and ferritin in patients with AOSD. The results are shown in **Figure 2**. Serum levels of Gal-3 (**Figure 2A**) were significantly and positively correlated with the Pouchot score and serum ferritin and IL-18 levels (all p <0.001). However, no significant correlation was observed between serum M2BPGi levels and AOSD disease activity score (**Figure 2B**). There were no significant correlations between CRP, a common inflammatory marker, and the Pouchot Score and ferritin (**Supplementary Figure 1**).

**Figure 3:**
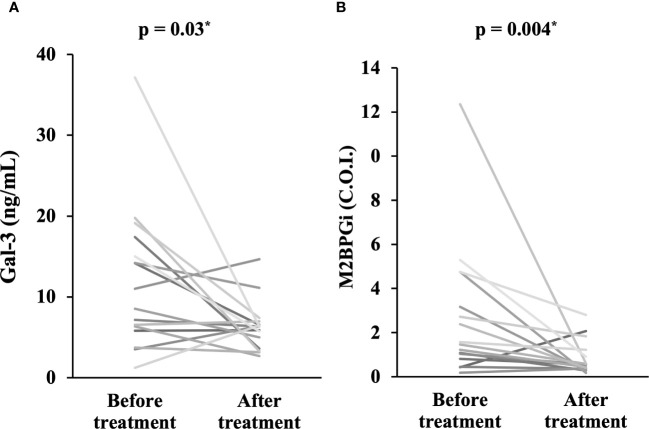
Comparison of serum levels of Gal-3 and M2BPGi before and after treatment. Comparisons of serum concentrations of **(A)** Gal-3 and **(B)** M2BPGi before and after treatment are shown. Both Gal-3 and M2BPGi are significantly lower after treatment compared to before treatment. Gal-3, galectin-3; M2BPGi, Mac-2 binding protein glycosylation isomer; C.O.I., cut off index. * means there is a significant difference at p <0.05.

To explore longitudinal changes in Gal-3 and M2BPGi, we included 16 patients with two longitudinal samples (at least 1 month apart). In the longitudinal study, 16 patients with active AOSD were followed up until disease activity was controlled and were then resampled. Serum levels of Gal-3 (**Figure 3A**) and M2BPGi (**Figure 3B**) significantly decreased along with ferritin levels after immunosuppressive treatment (p = 0.03 and 0.004, respectively).

### 3.3 Serum levels of Gal-3 and M2BPGi among AOSD disease phenotypes

**Figure 4:**
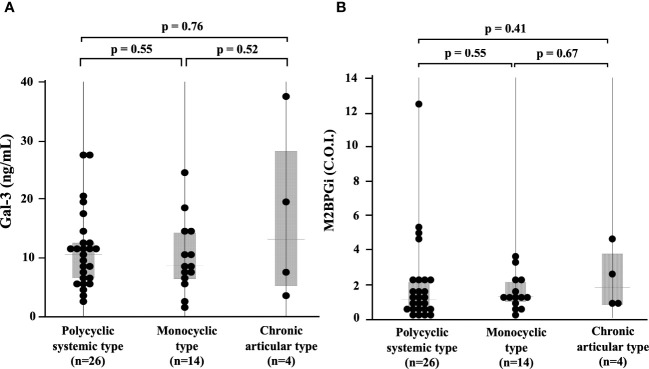
Comparisons of serum Gal-3 and M2BPGi concentrations in different phenotypes of AOSD. Comparisons of serum **(A)** Gal-3 and **(B)** M2BPGi concentrations by the phenotypes of AOSD are shown. There are no significant differences in serum levels of Gal-3 and M2BPGi among the phenotypes. Gal-3, galectin-3; M2BPGi, Mac-2 binding protein glycosylation isomer; C.O.I., cut off index.

**Figure 5:**
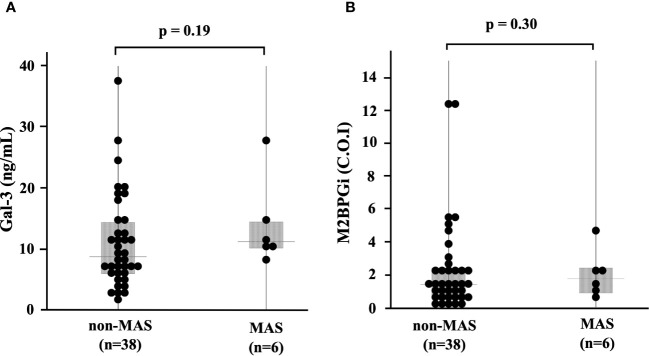
Serum levels of Gal-3 and M2BPGi in AOSD patients with and without MAS. Comparisons of serum **(A)** Gal-3 and **(B)** M2BPGi with and without MAS are shown. There are no significant differences in serum levels of Gal-3 and M2BPGi between AOSD patients with and without MAS. Gal-3, galectin-3; M2BPGi, Mac-2 binding protein glycosylation isomer; MAS, macrophage activation syndrome; C.O.I., cut off index.

**Figure 6:**
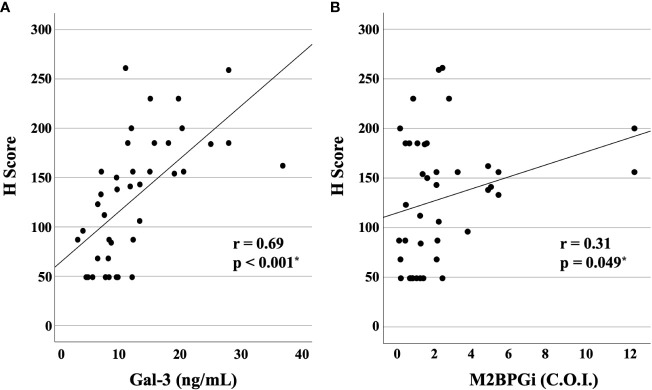
Correlations of serum levels of Gal-3 and M2BPGi or the reactive hemophagocytic syndrome diagnostic score (H score) for MAS. The correlations between serum **(A)** Gal-3 and **(B)** M2BPGi in AOSD patients and the H-score, a predictive score for MAS, are shown. There is a statistically significant positive correlation between Gal-3 and M2BPGi and H-scores. Gal-3, galectin-3; M2BPGi, Mac-2 binding protein glycosylation isomer; MAS, macrophage activation syndrome; C.O.I., cut off index. * means there is a significant difference at p <0.05.

We compared the serum levels of Gal-3 and M2BPGi before treatment among the three AOSD phenotypes (polycyclic systemic, monocyclic, and chronic articular). There were no significant differences in serum levels of Gal-3 and M2BPGi among these three AOSD phenotypes (**Figure 4**). Serum levels of Gal-3 and M2BPGi were elevated in AOSD patients with MAS, similarly, there were no significant differences in the serum levels of Gal-3 or M2BPGi between patients with AOSD with and without MAS (**Figure 5**). However, a statistically significant correlation was observed between the serum levels of Gal-3 (**Figure 6A**) but not M2BPGi (**Figure 6B**) or the H score in patients with AOSD (Gal-3: r = 0.69, p <0.001; M2BPGi: r = 0.31, p = 0.049).

### 3.4 Cytokine networks in patients with AOSD

**Figure 7:**
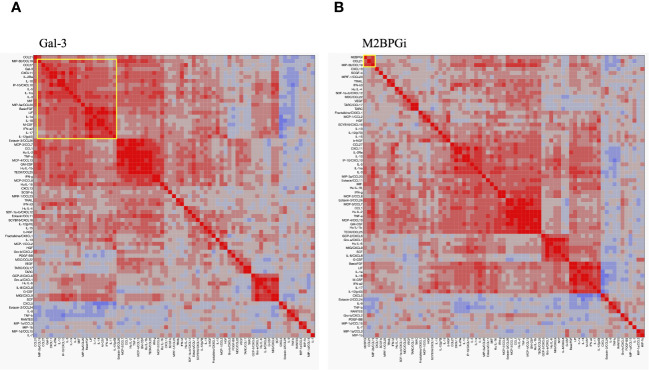
Cytokine networks in the patients with AOSD. Hierarchical clustering with a Pearson correlation heatmap of serum cytokine levels in patients with AOSD. Pearson correlation heat maps of Gal-3 **(A)** and M2BPGi **(B)** with cytokines are shown. The yellow-circled areas indicate clusters in which each cytokine is strongly correlated with the other. The major clusters correlated with Gal-3 and M2BPGi are designated as Cluster 1 and Cluster 2, respectively. AOSD, adult-onset Still’s disease; Gal-3, galectin-3; M2BPGi, Mac-2 binding protein glycosylation isomer.

To compare the cytokine networks between Gal-3 or M2BPGi and inflammatory cytokines in AOSD, we examined the correlations between Gal-3 or M2BPGi and the serum levels of activated individual cytokines in patients with AOSD. Hierarchical clustering with heat maps based on the Pearson correlation coefficients is shown in **Figure 7A** (for Gal-3) and **Figure 7B** (for M2BPGi). One major cluster (Cluster 1) of Gal-3 positively correlated with various inflammatory cytokines was identified. Cluster 1 showed significant correlations between Gal-3 and basic FGF (r = 0.70, p <0.0001), LIF (r = 0.66, p <0.0001), CCL27 (r = 0.65, p <0.0001), CXCL11(r = 0.65, p <0.0001), IL-1Ra (r = 0.63, p <0.0001), IL-2Ra (r = 0.63, p <0.0001), IL-3 (r = 0.62, p <0.0001), M-CSF (r = 0.58, p <0.0001), IL-18 (r = 0.58, p <0.0001), IFN-a2 (r = 0.53, p <0.0001), MIP-3b/CCL19 (r = 0.53, p = 0.0002), and IP-10/CXCL10 (r = 0.50, p = 0.0006) in patients with AOSD (**Table 2A**). In the M2BPGi clustering heatmaps, there were correlations between M2BPGi and some inflammatory cytokines (**Figure 7B**, Cluster 2). However, these correlations were not as strong as those of Gal-3 (**Table 2B**).

Table 2ACorrelations between serum cytokines and Gal-3 in AOSD patients (after Bonferroni`s correction).

**Table:** 

Cytokines	Correlation coefficient (95%CI)	p value
basic FGF	0.70 (0.51–0.82)	<0.0001^*^
LIF	0.66 (0.45–0.80)	<0.0001^*^
CCL27	0.65 (0.44–0.79)	<0.0001^*^
CXCL11	0.65 (0.44–0.79)	<0.0001^*^
IL-1Ra	0.63 (0.41–0.78)	<0.0001^*^
IL-2Ra	0.63 (0.40–0.78)	<0.0001^*^
IL-3	0.62 (0.39–0.77)	<0.0001^*^
M-CSF	0.58 (0.35–0.75)	<0.0001^*^
IL-18	0.58 (0.34–0.75)	<0.0001^*^
IFN-a2	0.53 (0.28–0.72)	0.0002^*^
MIP-3b/CCL19	0.53 (0.27–0.71)	0.0002^*^
IP-10/CXCL10	0.50 (0.24–0.69)	0.0006^*^
IL-12(p40)	0.47 (0.20–0.67)	0.0013
IL-10	0.47 (0.20–0.67)	0.0013
IL-1α	0.47 (0.20–0.67)	0.0014
MIF	0.41 (0.13–0.63)	0.0057
IL-5	0.40 (0.12–0.62)	0.0068
MIP-3a/CCL20	0.40 (0.11–0.62)	0.0075
IL-17	0.26 (–0.04 to 0.52)	0.0878

Gal-3; galectin-3, Basic FGF; basic fibroblast growth factor, LIF; leukemia inhibitory factor, CCL; chemokine (C-C motif) ligand, CXCL; chemokine (C-X-C motif) ligand, IL; interleukin, Ra; receptor antagonist, M-CSF; macrophage colony-stimulating factor, IFN; interferon, MIP; macrophage inflammatory protein, IP; interferon γ-induced protein, MIF; macrophage migration inhibitory factor.

*means there is a significant difference at p <0.001.

**Table:** TABLE 2B Correlations between serum cytokines and M2BPGi in patients with AOSD (after Bonferroni`s correction).

Cytokines	Correlation coefficient (95%CI)	p value
MIP-3b/CCL19	0.67 (0.44–0.82)	<0.0001^*^
CCL21	0.50 (0.19–0.71)	0.003

M2BPGi; mac-2 binding protein glycosylation isomer, MIP; macrophage inflammatory protein, CCL; chemokine (C-C motif) ligand.

*means there is a significant difference at p <0.001.

## 4 Discussion

In this study, we determined whether serum levels of Gal-3 or M2BPGi were linked to the inflammatory processes of AOSD. We demonstrated that both Gal-3 and M2BPGi were elevated in patients with AOSD; however, circulating Gal-3, but not M2BPGi, was highly correlated with AOSD disease activity (Pouchot score). Our study also revealed that the serum Gal-3 levels were highly correlated with the serum IL-18 levels, which are presumed to be useful biomarkers for autoinflammatory diseases, such as AOSD, systemic juvenile idiopathic arthritis, and NLRC4 inflammasomopathies (21, 22). The fact that circulating Gal-3 was correlated with various cytokines suggests that Gal-3 may contribute to the inflammatory cytokine network involved in the pathogenesis of AOSD. This finding is in line with previous reports showing that Gal-3 plays a role in the inflammatory process in immune-mediated disorders (23). Taken together, our and previous studies suggest a critical role for Gal-3 in AOSD, in addition to various immune-mediated disorders.

M2BP was identified as a ligand of Gal-3, a ubiquitous and multifunctional secreted glycoprotein (24). More than 300 proteins form complexes with Gal-3 in hematopoietic stem cells and peripheral blood mononuclear cells (25), and Gal-3 has been implicated in various inflammatory conditions (26). Targeting Gal-3 in mice resulted in decreased expression of Th1 and Th17 helper cells and reduced the severity of inflammation in inflammatory models (27). These observations suggested a pathogenic role for Gal-3 in inflammatory diseases. Previous studies have shown increased levels of circulating Gal-3 in autoimmune diseases, such as systemic lupus erythematosus, Behçet’s disease, and RA (28–30). In addition, plasma Gal-3 levels are elevated in patients with AOSD, in parallel with disease activity and inflammasome downstream cytokines (31). Our results are consistent with those of previous studies. In addition, we demonstrated that both Gal-3 and M2BPGi were elevated in AOSD, but only Gal-3 was significantly correlated with AOSD activity linked to the inflammatory cytokine network. In contrast, we found no significant correlations between M2BPGi and AOSD disease activity scores or serum levels of IL-18. Although circulating M2BPGi may reflect various macrophage activation statuses by interacting with Gal-3 (2), M2BPGi-positive macrophages might have a more profound impact on the activation of macrophages *via* Gal-3 (32). Although there was no significant correlation between the serum levels of Gal-3 and M2BPGi (data not shown), it is possible that the cross-talk between M2BPGi-positive macrophages and Gal-3 may be involved in macrophage activation status in AOSD.

The exact mechanism by which Gal-3 is increased in patients with active AOSD remains unknown; however, several possibilities have been hypothesized. Previous studies have uncovered that Gal-3 can stimulate the expression and secretion of many pro‐inflammatory factors, including IL‐6 and TNF‐α by activating monocyte or macrophages (33). Nucleotide-binding oligomerization domain-like receptor family, pyrin domain-containing 3 (NLRP3) can be activated by danger-associated molecular patterns (DAMPs) and Gal-3 is considered a DAMP molecule (34). Specific binding of Gal-3 to NLRP3 and inflammasome activation were shown in a mice model of primary biliary cholangitis (35). Although the pathogenesis of the disease is not fully elucidated, inflammasome activation is considered to be involved in the disease pathogenesis of AOSD (11). Our results also demonstrated that serum levels of Gal-3 were highly correlated with serum levels of IL-18, which is processed to its mature form by activated inflammasomes. Taken together, our data suggest that increased Gal-3 levels are implicated in the autoinflammatory cascade of AOSD by activating inflammasomes. However, the complex biology of Gal-3, including its various interactions and signaling cascades, makes it difficult to elucidate its mechanism of action.

Furthermore, Gal-3 was significantly correlated with the H Score; this suggests that Gal-3 may be useful as a predictor of MAS as well as disease activity. Determining the H Score is invasive and costly, because it requires confirmation of organ enlargement through imaging and of hemophagocytosis through bone marrow examination (17). Gal-3, in contrast, can be measured by blood testing alone and may be a cheaper and simpler alternative.

In our study, M2BPGi clustered with only MIP-3β/CCL19 and CCL21. CCL19 and CCL21 are important regulators of continuous immunosurveillance; homeostasis; and induction of T cell activation, immune tolerance, and inflammatory responses during development (36). M2BPGi is poorly associated with cytokines contributing to the macrophage activation status, which is presumed to be involved in AOSD pathogenesis; instead, it appears to be correlated with the chemokines associated with immune system cell recruitment and cell–cell interactions. CCL19 and CCL21 are expressed on macrophages and synovial fibroblasts and have also been reported to be elevated in the sera of patients with chronic inflammatory diseases, such as RA and atherosclerosis (37, 38). The reason for the association between M2BPGi and these chemokines is currently unclear; however, M2BPGi and these chemokines have been implicated in chronic inflammation and progressive fibrosis in the liver and may reflect hypercytokinemia-associated liver damage (6, 39) Further studies are required to elucidate the association of M2BPGi with these chemokines.

Gal-3 was associated with multiple cytokines. Based on the existing AOSD cytokine cluster analysis, Gal-3 appears to be associated with the cytokines involved in CD4+ T cell development, activation, or function (including interferon alpha-2, basic FGF, IL-2, and IL-3) and with the cytokines involved in the cytokine release syndrome (including IL-18, CXCL10, M-CSF, and IL-1Ra) (40, 41). Furthermore, basic FGF is considered a useful diagnostic biomarker for AOSD (42), and Gal-3 showed the strongest correlation with basic FGF. Thus, Gal-3 is elevated in AOSD, which has high disease activity and is prone to causing the cytokine release syndrome; accordingly, it may be a useful biomarker for the prediction of MAS.

There are several similarities between the clinical manifestations and cytokine profiles of patients with coronavirus disease-2019 and AOSD (43). Cytokine production is dysregulated in response to severe acute respiratory syndrome coronavirus 2 (SARS-CoV-2) infection and cytokine production is dysregulated, leading to systemic hyperinflammation or a cytokine storm in severe cases (44). Both patients with active AOSD or with severe coronavirus disease-2019 showed elevated Gal-3 and cytokines, including IL-1β, IL-6, and IL-18, supporting a common link of cytokine storm in their pathogenesis (45). Several recent studies have identified a direct correlation between the circulating levels of Gal-3 and the severity of SARS-CoV-2 infection (46), suggesting a role of Gal-3 in the cytokine storm observed in SARS-CoV-2 infections. These findings suggest that Gal-3 plays a role in the inflammatory cytokine cascades or cytokine storm seen in severe SARS-CoV-2 infections as well as in patients with AOSD.

The present study had some limitations. First, this was a cross-sectional study. Prospective studies are needed to elucidate the association between Gal-3 and AOSD progression or prognosis. Second, the study population comprised only Japanese people; therefore, it is not clear whether the findings of this study can be applied to other ethnic groups. Third, multiple serum cytokines were measured in patients with AOSD but not in control patients with RA or healthy participants. In addition, the number of patients with AOSD included in the analysis was small. Further studies with a larger number of cases and innate immune cellular analyses are needed to elucidate the mechanisms underlying the elevation of Gal-3.

In conclusion, we measured Gal-3 and M2BPGi levels in patients with AOSD and demonstrated that these molecules’ levels were significantly elevated in patients with AOSD and were downregulated by immunosuppressive treatments. Furthermore, serum levels of Gal-3 were significantly correlated with disease activity, such as Pouchot score or serum levels of ferritin, and H-score, a predictive score for MAS, as well as inflammatory cytokines, including IL-18, in patients with AOSD. Thus, we believe that Gal-3 may be an effective biomarker for the prediction of disease activity and cytokine release syndrome, including MAS, in AOSD and may provide useful information concerning disease activity and inflammatory cytokine cascades in patients with AOSD.

## Data availability statement

The original contributions presented in the study are included in the article/**Supplementary Material**. Further inquiries can be directed to the corresponding author.

## Ethics statement

The studies involving humans were approved by Institutional Review Board of Fukushima Medical University. The studies were conducted in accordance with the local legislation and institutional requirements. The participants provided their written informed consent to participate in this study.

## Author contributions

SY: Conceptualization, Data curation, Formal analysis, Investigation, Methodology, Software, Visualization, Writing – original draft, Writing – review & editing. TK: Data curation, Formal analysis, Resources, Software, Supervision, Validation, Visualization, Writing – review & editing. YF: Validation, Data curation, Investigation, Supervision, Writing – review & editing. HY: Data curation, Formal analysis, Resources, Software, Supervision, Validation, Writing – review & editing. HM: Data curation, Investigation, Validation, Writing – review & editing. YS: Data curation, Writing – review & editing. KS: Data curation, Writing – review & editing. SS: Data curation, Writing – review & editing. TA: Data curation, Writing – review & editing. MK: Investigation, Supervision, Validation, Writing – review & editing. HO: Investigation, Supervision, Validation, Writing – review & editing. MM: Supervision, Validation, Writing – review & editing. MS: Data curation, Investigation, Methodology, Resources, Supervision, Validation, Writing – review & editing. KM: Methodology, Project administration, Supervision, Validation, Visualization, Writing – original draft, Writing – review & editing, Conceptualization, Data curation, Funding acquisition, Investigation.
