# Supplementary figures and images for: Serum Mac-2 binding protein glycosylation isomer and galectin-3 levels in adult-onset Still’s disease and their association with cytokines
Source: Front Immunol. 2024 Apr 22;15:1385654. doi: 10.3389/fimmu.2024.1385654 (PMC11073344; doi:10.3389/fimmu.2024.1385654)

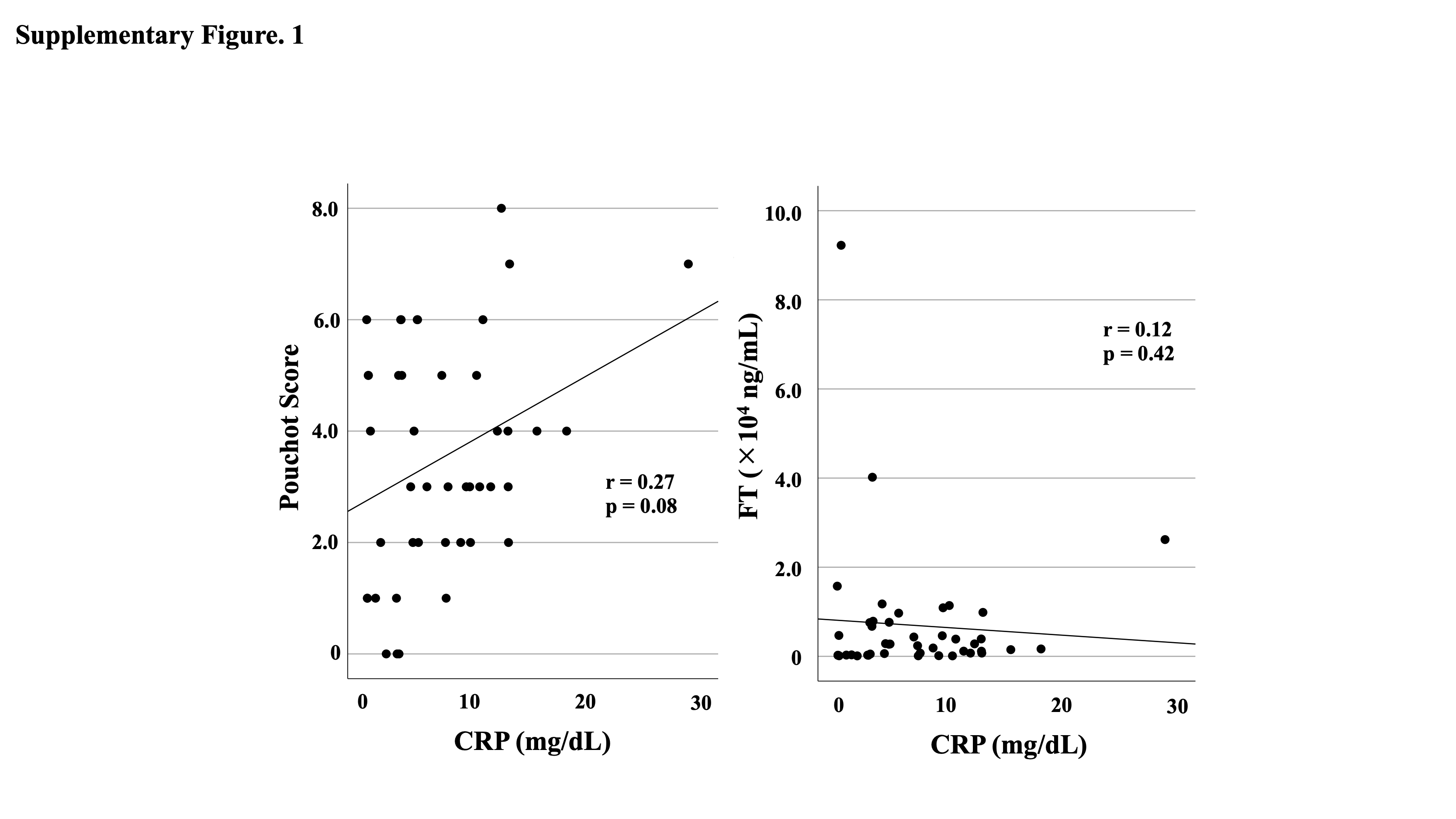

Supplement: Supplementary file 1 [file Image_1.tiff]
